# Natural language processing (NLP) tools in extracting biomedical concepts from research articles: a case study on autism spectrum disorder

**DOI:** 10.1186/s12911-020-01352-2

**Published:** 2020-12-30

**Authors:** Jacqueline Peng, Mengge Zhao, James Havrilla, Cong Liu, Chunhua Weng, Whitney Guthrie, Robert Schultz, Kai Wang, Yunyun Zhou

**Affiliations:** 1 School of Engineering and Applied Science, University of Pennsylvania, Philadelphia, PA 19104 USA; 2 Raymond G. Perelman Center for Cellular and Molecular Therapeutics, Children’s Hospital of Philadelphia, Philadelphia, PA 19104 USA; 3 Department of Biomedical Informatics, Columbia University, New York, NY 10032 USA; 4 Center for Autism Research, Children’s Hospital of Philadelphia, Philadelphia, PA 19104 USA; 5 Department of Psychiatry, Perelman School of Medicine, University of Pennsylvania, Philadelphia, PA 19104 USA; 6 Department of Pathology and Laboratory Medicine, University of Pennsylvania, Philadelphia, PA 19104 USA

**Keywords:** Natural language processing, Machine learning, Named entity recognition, Autism spectrum disorder

## Abstract

**Background:**

Natural language processing (NLP) tools can facilitate the extraction of biomedical concepts from unstructured free texts, such as research articles or clinical notes. The NLP software tools CLAMP, cTAKES, and MetaMap are among the most widely used tools to extract biomedical concept entities. However, their performance in extracting disease-specific terminology from literature has not been compared extensively, especially for complex neuropsychiatric disorders with a diverse set of phenotypic and clinical manifestations.

**Methods:**

We comparatively evaluated these NLP tools using autism spectrum disorder (ASD) as a case study. We collected 827 ASD-related terms based on previous literature as the benchmark list for performance evaluation. Then, we applied CLAMP, cTAKES, and MetaMap on 544 full-text articles and 20,408 abstracts from PubMed to extract ASD-related terms. We evaluated the predictive performance using precision, recall, and F1 score.

**Results:**

We found that CLAMP has the best performance in terms of F1 score followed by cTAKES and then MetaMap. Our results show that CLAMP has much higher precision than cTAKES and MetaMap, while cTAKES and MetaMap have higher recall than CLAMP.

**Conclusion:**

The analysis protocols used in this study can be applied to other neuropsychiatric or neurodevelopmental disorders that lack well-defined terminology sets to describe their phenotypic presentations.

## Background

The extraction of biomedical concepts and entities, such as genes, drugs, and symptoms, is one of the initial steps for many natural language processing (NLP) analyses. It constitutes a named-entity recognition (NER) task tailored to the biomedical domain. Three popular biomedical information extraction tools are MetaMap [1], cTAKES [2], and CLAMP [3]. Common to these tools is a biomedical NER feature based on Unified Medical Language System (UMLS) concepts, which represents a standardized and comprehensive biomedical vocabulary [4], that uses dictionary-lookup and machine learning approaches. MetaMap was published in 2001 and is considered the foundational biomedical information extraction tool developed by the National Library of Medicine. cTAKES was later developed by Mayo Clinic in 2010 and included more NLP functional modules to process clinical notes using rule-based and machine learning-based approaches. Compared to the other two, the recently developed NLP tool CLAMP has a greater emphasis on flexibility in the development of customized pipeline tasks with diverse options for information extraction.

Previous studies comparing MetaMap, cTAKES, and CLAMP on electronic health record (EHR) clinical notes have been published. Reátegui et al. compared the performances of MetaMap and cTAKES on NER tasks and found that cTAKES is slightly better in analyzing clinical notes [5]. In addition, the CLAMP team compared their tool to the two others and concluded that CLAMP outperforms them in analyzing clinical notes [3]. However, there is a need for an unbiased third-party evaluation of these tools, particularly on corpora other than clinical notes, for example, PubMed research articles. Biomedical literature holds a wealth of information on disease, genomic, phenotypic information, and their relationships, and there is a tremendous growth in effort to mine these unstructured texts to gain insights about diseases [6]. To take advantage of the vast amount of published scientific literature in learning disease-phenotype relationships automatically, it is necessary to formulate best practices to extract such knowledge from research articles. This is especially important for complex neuropsychiatric disorders with a diverse set of phenotypic and clinical manifestations, as these disorders do not have a well-recognized and widely accepted terminology/vocabulary set. Here we evaluated the three tools in extracting biomedical entities from literature using autism spectrum disorder (ASD) as a case study.

ASD is a complex neurodevelopmental disorder that affects 1 in 59 children in the United States [7, 8]. Diagnosing and characterizing ASD can be very difficult, as patients with ASD have markedly heterogeneous presentations of the core ASD symptom domains (i.e. social interaction, communication, and restricted and repetitive behavior). However, few ASD phenotype terminology sets exist that can assist with well-defined, comprehensive studies of ASD. Other complex neuropsychiatric disorders like schizophrenia also face similar issues in defining their terminologies [9]. Therefore, ASD is an apt disorder on which to test the performance of NLP tools in recognizing disease-specific vocabulary. The insights gained from studying ASD could be applied to other challenging diseases, facilitating future therapeutic development and precision medicine.

In the present study, we compared the performance of CLAMP, cTAKES, and MetaMap in extracting ASD-specific terminology from PubMed full-text journal articles and abstracts. The NER component of each the three tools were used to extract biomedical concepts and entities from these unstructured texts; the assumption is that these tools will be able to extract ASD-related vocabulary when applied on PubMed full-texts and abstracts in the ASD domain. We used a previously published ASD terminology set as a benchmark against which to compare the three tools. We also evaluated the possibility of using the terms extracted by these tools to build a more comprehensive list of ASD terminology. Through this analysis, we hope to provide some insight into how these tools can be best used in the future to aid ASD characterization and diagnosis.

## Methods

### Retrieval of ASD-related PubMed full-text articles and abstracts

PubMed abstracts were retrieved based on MeSH Major Topic using the search query: “Autism Spectrum Disorder”, “Autistic Disorder”, “Asperger syndrome”, and “Spectrum Disorders, Autism”. To make the topics of articles more relevant to the clinical problems of ASD, we only kept PubMed full-texts related to ASD children’s communication, ASD behaviors, interpersonal relations of ASD individuals, and ASD psychologies based on the classification of APA PsycNet (https://psycnet.apa.org/) followed by manual review. A total of 544 full-text articles and 20,408 PubMed abstracts were used for downstream analysis.

### Benchmark ASD terms and rule-based labelling approach

Because we wanted to analyze a large volume of PubMed full-text articles and abstracts, we felt that it was not feasible to generate gold standard labels of ASD entities manually and wanted to instead use an automatic rule-based labelling approach. Therefore, as a starting point, we collected a total of 821 distinct ASD-related terms, extracted from clinical notes, published by Lingren et al. [10] and derived from Barbaresi et al. [11], which is the only published and freely available ASD terminology set as far as we know. These terms, however, do not represent a comprehensive set of ASD vocabulary. We additionally added the following six ASD terms manually, with their respective UMLS Concept Unique Identifier (CUI), to form a set of 827 unique ASD-related terms: “Autism” (C0004352), “Autism Spectrum Disorder” (C1510586), “Autistic” (C0004352), “ASD” (C1510586), “ASDs” (C1510586), and “Asperger” (C0236792). These 827 terms were considered to be our benchmark (BM) set of ASD terms used to label entities in the PubMed full-text articles and abstracts as true entities. These terms can be found in Additional file 3: Table S1. We certainly acknowledge that higher quality terminology sets for ASD exist, but there is a substantial license fee to use these terminology sets, which is a problem that we wish to address in the future by releasing open-access terminology sets. First, we tokenized the 544 full-text PubMed articles and 20,408 abstracts using spaCy version 2.2.1 (https://spacy.io/), a Python library for natural language processing. After tokenization, exact matches on the terms in the BM set were performed using spaCy, using the longest match possible in the BM set (i.e. “autism spectrum disorder is characterized by…” is labelled with “autism spectrum disorder” instead of “autism”). Matching was case-insensitive for all BM terms except for “ASD” and “ASDs” which are case-sensitive. Each match was considered a true entity. We acknowledge that a limitation of using the BM set and using a rule-based labelling approach is that not all ASD-related terminology is captured. However, it overcame the difficulty, cost, and time-consuming nature of a manual-labelling process, especially in annotating the high volume of full-texts (544) and abstracts (20,408) that we used.

### Extraction of entities using CLAMP, cTAKES, and MetaMap

CLAMP, cTAKES, and MetaMap were used to extract entities from the ASD-related PubMed full-texts and abstracts. CLAMP version 1.6.1 was used and the pipeline used for the CLAMP analysis was the built-in default "clamp-ner-attribute" pipeline. For the "DF_Dictionary_based_UMLS" component of the pipeline, the "Adjust Named Entity Offset or not?" option was unchecked and the "UMLS source" option was set to "ALL" in order to map as many terms as possible to UMLS. Default options were used for all other parameters. For the cTAKES (version 4.0.0) analysis, the built-in "Default Clinical Pipeline" was used with all default options. We then used the ctakes-parser Python package (https://pypi.org/project/ctakes-parser/ version 0.1.0) to transform the cTAKES output from .xmi format to.csv format. We used MetaMap 2018 to analyze the data, with the UMLS database in version 2018AB. All default options were used for the UMLS mapping and additionally the word sense disambiguation option was used. MetaMap only recognizes ASCII characters, so we converted non-ASCII characters to ASCII and removed the unconvertable characters in the full-texts and abstracts before inputting them into MetaMap. MetaMap outputs results in XML format, splitting each text input into multiple sections. We recognize that this behavior caused some difficulty in extracting back the original full-texts and abstracts, which resulted in the loss of some true entities.

### Semantic type filtering and comorbid psychiatric disorder filtering

For the baseline results, the predicted entities outputted by CLAMP, cTAKES, and MetaMap were directly analyzed. However, we noticed that the precision of these tools was low due to the presence of predicted entities not specific to ASD, and therefore not present in the BM set of ASD terms. The 827 terms from the BM set represent 96 valid UMLS Concept Unique Identifiers (CUIs) and 13 unique semantic types. For these 827 terms, the two most prevalent semantic types were Finding (fndg, T033) and Mental or Behavioral Dysfunction (mobd, T048), respectively. We also think these two semantic types are most relevant to ASD phenotypes and clinical manifestations. Therefore, we used these two semantic types to filter the predicted entities outputted by CLAMP, cTAKES, and MetaMap such that only predicted entities falling under the two types were kept. However, we kept all entities mapping to the CUI for Atrial Septal Defects (C0018817), since the tools may not be able to disambiguate its abbreviation “ASD”, which it shares with autism spectrum disorder; the CUI falls under the semantic type of Congenital Abnormality (T019), and would be filtered out. In order to filter by semantic type, only predicted entities with valid CUIs were used (i.e. the CUI has a length of 8 characters and starts with “C”). Finally, psychiatric comorbidities were filtered out; a list of terms and CUIs representing comorbid psychiatric disorders in individuals with ASD (Additional file 3: Table S2) was generated based on a paper by Leyfer et al. [12]. Predicted entities mapping to the same CUI of these comorbidity terms were filtered out.

### Performance statistics

The prediction of ASD-related entities in full-text articles and abstracts is a named entity recognition (NER) task. True entities are labelled by the rule-based matching approach described in the “Benchmark ASD terms and rule-based labelling approach” section. A true positive is counted when, for a given article or abstract, a predicted entity overlaps in position with a true entity on at least one character. We thought a relaxed match was more appropriate than a strict match because of the limitations of the BM terms and the rule-based labelling method. Only the one true entity is counted if there are multiple overlapping predicted entities. Therefore, the total number of true positives is the total number of true entities with an overlapping predicted entity. The precision is the total number of true positives divided by the total number of predicted entities. The recall is the total number of true positives divided by the total number of true entities. The F1 score is calculated as $\frac{2\times precision\times recall}{precision+recall}$.

## Results

### Identifying the most frequent ASD terms in PubMed literature

To conduct the current study, we compiled 544 full-text articles and 20,408 abstracts from PubMed that are relevant to ASD phenotypes. We also built a benchmark (BM) set of 827 ASD terms to label entities in the PubMed full-text articles and abstracts as true entities; these terms include 821 distinct ASD-related terms published by Lingren et al. [10], which is the only published and freely available ASD terminology set that we know, together with 6 additional terms added by us (see “Methods”). These additional ASD terms were added to increase the comprehensiveness of the set, since they denote ASD as a disorder. Initially, we examined which terms from the BM ASD set were the most frequent in the PubMed full-text articles and abstracts. A total of 48,706 BM entities representing 96 unique (case-insensitive) BM terms were extracted from the 544 full-text articles (Additional file 1: Figure 1A). Terms describing ASD as a disorder, such as “ASD”, “autism”, and “autistic”, were the most common. Among the terms related to general characteristics of ASD, “nonverbal/non-verbal”, “imitation”, and “reciprocity” were the most common. The 96 unique full-text BM terms encompass 48 unique UMLS Concept Unique Identifiers (CUI) and 10 unique UMLS semantic types. In addition, a total of 106,284 BM entities representing 106 unique (case-insensitive) BM terms were extracted from the 20,408 PubMed abstracts (Additional file 1: Figure 1B). Similar to the full-texts, terms describing ASD as a disorder were the most common, and among the terms related to general characteristics of ASD, “imitation”, “nonverbal/non-verbal”, “language delay”, and “reciprocity” were the most frequent. The 106 unique abstract BM terms encompass 52 unique CUI and the same 10 semantic types as the full-texts.

### CLAMP showed higher F1 score than cTAKEs and MetaMap on full-texts and abstracts

**Fig. 1:**
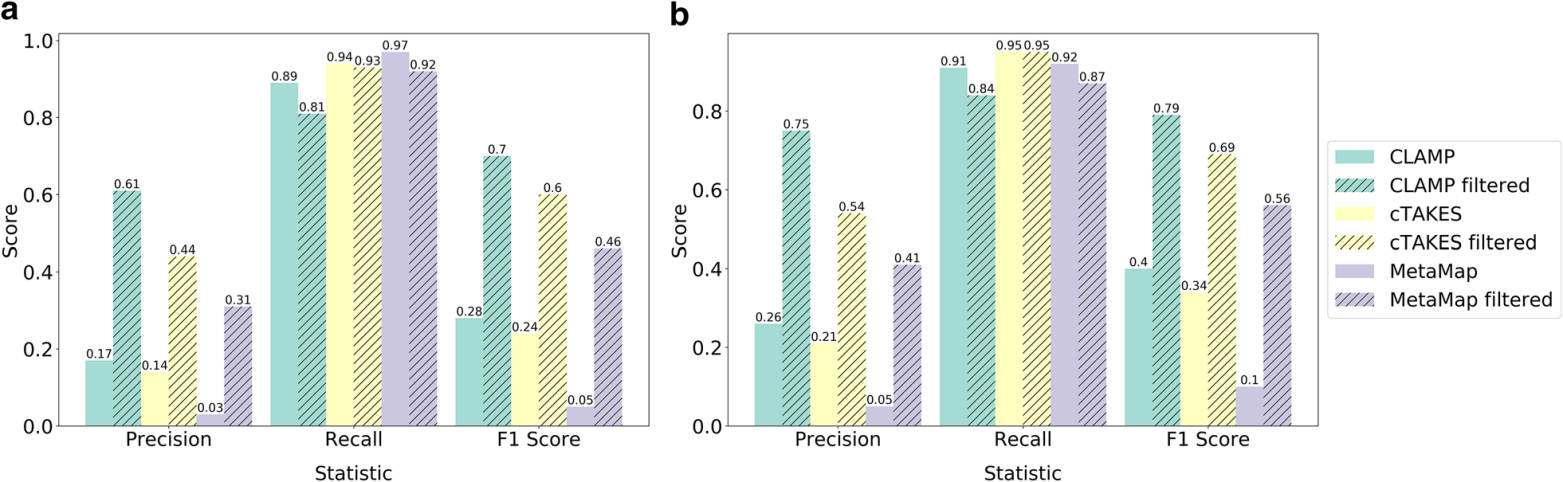
Performance comparison of CLAMP, cTAKES, and MetaMap. Shown here are the performance statistics (precision, recall, and F1 score) of the three tools in extracting ASD terms from (**a)** 544 PubMed full-text articles and (**b)** 20,408 PubMed abstracts. Using a rule-based matching approach, a benchmark set of ASD terms was used to label what was considered to be the true entities in the texts. A true entity counts as a true positive if a predicted entity (from CLAMP, cTAKES, or MetaMap) overlaps with the true entity. The precision is the number of true positives divided by the total number of predicted entities (by one of the three tools). The recall is the number of true positives divided by the total number of true entities. The F1 score is calculated as (2 × precision × recall)/(precision + recall). The solid bars represent the results when using the unprocessed predictions from the three tools, and the hatched bars represent the results when first filtering the predicted entities according to the process described in “Methods”

The entities labelled using the BM ASD terms, treated as the true entities, were compared to the entities predicted by CLAMP, cTAKES, and MetaMap. The precision, recall, and F1 score for each tool are detailed in Table 1 when tested on the 544 full-text PubMed articles and Table 2 when tested on the 20,408 PubMed abstracts. Overall, we found that CLAMP has the best performance in terms of F1 score, followed by cTAKES, and then MetaMap, for both the baseline result and when filtering the predicted entities by UMLS semantic type and removing comorbid psychiatric disorders. This result is consistent when using either the PubMed full-texts or abstracts. The overview of performance comparison can be found in Fig. 1. CLAMP’s best performance is largely due to the fact that it predicts less false positive (FP) entities than cTAKES and MetaMap, resulting in higher precision. However, both MetaMap and cTAKES have a higher recall than CLAMP. There was also a notable increase in performance when the predicted entities were filtered to keep only the semantic types Finding (T033) and Mental or Behavioral Dysfunction (T048), and filtered to remove ASD psychiatric comorbidities, such as attention deficit hyperactivity disorder (ADHD) and anxiety. Using the PubMed abstracts instead of the full-texts also increased the performance of all three tools. This is expected, since the abstracts generally contain more ASD-specific information and therefore produce greater precision values when being analyzed by the tools. We should also stress here that since the BM set of ASD terms are not comprehensive, the F1 scores for all methods are generally low (compared to F1 scores reported in other similar studies), implicating the substantial challenges and the large room for improvements in NLP analysis of complex psychiatric disorders such as ASD.

**Table 1:** Precision, recall and F1 scores of CLAMP, cTAKES, and MetaMap on 544 ASD-related full-text PubMed articles

	Number of true positives	Number of true entities	Number of predicted entities	Precision	Recall	F1 Score
CLAMP unfiltered	43,330	48,706	256,525	0.17	0.89	0.28
CLAMP filtered	39,533	48,706	65,037	0.61	0.81	0.70
cTAKES unfiltered	45,579	48,706	337,125	0.14	0.94	0.24
cTAKES filtered	45,509	48,706	103,783	0.44	0.93	0.60
MetaMap unfiltered	47,544	48,804	1,726,985	0.03	0.97	0.05
MetaMap filtered	45,078	48,804	145,926	0.31	0.92	0.46

The number of true entities represents the number of benchmark (BM) ASD terms found in the texts. MetaMap has a slightly different number of true entities than CLAMP and cTAKES because of the pre-processing methods used in order to run MetaMap on the texts. Details on how the statistics were computed can be found in “Methods”

**Table 2:** Precision, recall and F1 score of CLAMP, cTAKES, and MetaMap on 20,408 ASD-related PubMed abstracts

	Number of true positives	Number of true entities	Number of predicted entities	Precision	Recall	F1 Score
CLAMP unfiltered	96,235	106,284	370,654	0.26	0.91	0.4
CLAMP filtered	89,185	106,284	118,862	0.75	0.84	0.79
cTAKES unfiltered	101,219	106,284	489,520	0.21	0.95	0.34
cTAKES filtered	101,127	106,284	185,966	0.54	0.95	0.69
MetaMap unfiltered	97,992	106,286	1,839,606	0.05	0.92	0.10
MetaMap filtered	92,570	106,286	224,282	0.41	0.87	0.56

The number of true entities represents the number of benchmark (BM) ASD terms found in the texts. MetaMap has a slightly different number of true entities than CLAMP and cTAKES because of the pre-processing methods used in order to run MetaMap on the texts. Details on how the statistics were computed can be found in “Methods”.

The relatively low precision for all three tools, especially when analyzed without filtering methods, suggests that, while noise terms may be present in the predicted entities, there may also exist true ASD-related terms among the predicted entities that are not in the BM set. When examining the list of FP entities, we found that MetaMap is particularly noisy, predicting entities such as “used”, “found”, “related”, and “results”, under the semantic type of Finding (fndg, T033). MetaMap also predicts numerical entities under the same semantic type of Finding, which is not useful for the purposes of ASD phenotyping. For cTAKES, the top FP entities include “diagnosis”, “related”, and “test”, which represent generic terms. CLAMP FP entities include generic terms as well, such as “disorder” and “symptoms”. Altogether, these results implicate the need to filter out such generic terminology when using these tools to retrieve ASD-specific terminology from research articles.

### Analysis of predictions from CLAMP, cTAKES, and MetaMap

Because the BM set of ASD terms are not comprehensive, some FP predictions may be genuinely relevant to ASD. To address this issue, we attempted to narrow down the list of FP predictions outputted by CLAMP, cTAKES, and MetaMap. We only considered their output when run on the PubMed abstracts since there is likely more ASD-specific information in the abstracts than full-texts, which is the interpretation of the higher F1 scores. Furthermore, we used the filtered output rather than the raw output of the three tools to reduce the amount of noise in the predictions. Importantly, we only considered the CUIs of FP terms that appeared in a sentence with a general BM term (i.e. generalized ASD characteristics rather than terms like “ASD”, “autism”, and “autistic”, etc.), in order to retrieve characteristics of ASD, and where the CUI was predicted by all three tools in the sentence. We believed these filtering steps would consolidate the FP predictions to the most relevant terms. We consolidated the list of FP terms based on CUI instead of the terms themselves in order to capture variations of terms describing the same concept. The final prioritized list of FP CUIs and the corresponding terms mapped to them is listed along with the BM term they most frequently co-occur with in Additional file 3: Table S3. The prioritized CUIs and their frequencies among the FP predictions are also visualized in Additional file 2: Figure 2. Manual examination of these filtered FP predictions in the future, along with the sentences they appear in for context, is warranted.

When examining the predicted entities, we also found that CLAMP predicts the longest entities with the settings we used. The average number of words for an entity predicted by CLAMP, using the full-texts, is 2.30 ± 1.61 SD (2.26 ± 1.64 SD when filtered), the average for cTAKES is 1.11 ± 0.367 SD (1.15 ± 0.413 SD when filtered), and the average for MetaMap is 1.10 ± 0.339 SD (1.20 ± 0.472 SD when filtered). We can also examine predicted entities that overlap with the BM labelled entities to capture terms beyond what is provided by the limited BM set. As an example, Table 3 demonstrates BM labels with their respective overlapping CLAMP-predicted entities and the sentences and papers they appear in. The true positive predictions of the three tools, where the prediction contains a BM term, were aggregated by their overlapping BM term and can be found in Additional file 3: Tables S4 and S5 for full-texts and abstracts, respectively. Through this example, we see the possible utility of using NLP tools to expand on the current ASD terminology, given that the BM is clearly not a comprehensive set of ASD-relevant terminology. Furthermore, we can also examine the sentences where BM terms are found to better understand the context of their relationships to ASD and also to extract other ASD-related information.

**Table 3:** Overlapping entities between labelled benchmark ASD terms and CLAMP predictions

True entity	Predicted entity	Sentence	Full-text PubMed article
Speech and language delay	Severe speech and language delay	Patient 290,951 had a clinical diagnosis of autism spectrum disorder, behavioral difficulties, and *severe speech and language delay*	PMC5798319
Trains	A preoccupation with trains	Mr Parsons has a narrow range of specialist interests, including *a preoccupation with trains*, and also experiences a high degree of sensory sensitivity	PMC6394789
Limited eye contact	Poor and limited eye	We observed *poor and limited eye* contact in reciprocal social interactions during the ADOS examination	PMC5282903
Body rocking	Repeated incidents of body rocking	A case study with six children on the spectrum was conducted to observe *repeated incidents of body rocking*, hand flapping, and/or simultaneous body rocking and hand flapping	PMC5298619
Body rocking	Simultaneous body rocking	A case study with six children on the spectrum was conducted to observe repeated incidents of body rocking, hand flapping, and/or *simultaneous body rocking* and hand flapping	PMC5298619
Limited speech	Very limited speech output	Because these children had *very limited speech output* prior to treatment, the acquisition of speech sounds through AMMT is an important gain that provides a foundation for subsequent speech therapy	PMC3183050
Initiating social interactions	Difficulty initiating social interactions	Has *difficulty initiating social interactions* and demonstrates clear examples of atypical or unsuccessful responses to social overtures of others	PMC6055683
Trains	Toy trains	*Toy trains* appeared to be one of the most familiar and interesting toy for both ASD and TD children in the age range considered and was chosen as the non-social reward image	PMC5468258
Head banging	Unexpected head banging	Pitching into others with the head (violent and *unexpected head banging*, head against other's chest)	PMC3006199

The true entity represents the term from the benchmark set of ASD terms. These examples were chosen to illustrate how CLAMP’s predictions can take into account the context surrounding the benchmark terms, expanding the benchmark vocabulary. The entity predicted by CLAMP is italicized in the sentence

## Discussion

Autism spectrum disorder (ASD) is a challenging disorder to diagnose because of its heterogeneity in clinical manifestations [13, 14]. Therefore, the compilation of a set of comprehensive ASD terminology is needed to aid in the characterization and diagnosis of ASD. The clinical-based NLP tools CLAMP, cTAKES, and MetaMap can aid in the extraction of ASD phenotype terminology. In this study, we compared the performance of these tools in extracting ASD terminology, using a benchmark set of terms for evaluation, from full-text ASD research articles as well as abstracts.

We found that CLAMP has the best performance in terms of F1 score followed by cTAKES and then MetaMap, both when the tools are tested with full-texts and abstracts. This is largely due to the fact that CLAMP has much higher precision than cTAKES and MetaMap, as the entities it predicted are more disease problem focused. However, cTAKES and MetaMap have higher recall than CLAMP. We also found that filtering the predicted entities to only use the two most frequent semantic types, Finding (T033) and Mental or Behavioral Dysfunction (T048), as well as filtering out known psychiatric ASD comorbidities, increased the performance significantly. Furthermore, the performance of the NLP tools was better on abstracts than full-texts, likely due to more condensed ASD-specific information in abstracts. MetaMap and cTAKES apply a dictionary lookup approach that attempts to map noun phrases to UMLS; this is different than CLAMP, which uses a machine learning approach. In the context of research articles, the dictionary lookup may be less favorable in some circumstances since irrelevant non-ASD-related terms get mapped, creating slightly higher recall for MetaMap and cTAKES at the cost of largely lower precision relative to CLAMP. CLAMP and cTAKES, although tuned on clinical notes rather than biomedical literature, were shown to perform well in the later domain demonstrating flexibility in their use. Indeed, there are shared semantic characteristics between the domains of biomedical literature and clinical text, which suggests that techniques can be shared across the two domains [15]. Furthermore, the use of biomedical literature can be beneficial when there are challenges accessing sensitive clinical notes.

Besides the better performance, CLAMP has several additional advantages over other NLP tools. CLAMP has the ability to categorize predicted entities as a *problem*, *test*, and *treatment*, among other types, which allows for additional filtering and can help increase its precision (Additional file 3: Table S6). Another advantage of CLAMP is its ability to return the full-length predicted entity or adjust the length of the entity using a dictionary-based UMLS encoder. The ability to predict long chunks of text is possible owing to the machine learning approach CLAMP takes for NER instead of a dictionary lookup approach, which cTAKES and MetaMap take, that limits predictions to those in the dictionary and some variations. In many cases a longer phrase representing an entity would be more meaningful in characterizing ASD, for example, “non-verbal communication deficits” is more informative than “non-verbal”. One disadvantage of CLAMP was its lower recall than MetaMap and cTAKES, owing to the fewer amount of predictions made. The recall can be increased, however, by using CLAMP’s ability to perform case-insensitive and stemmed matching on a custom dictionary [3].

The FP terms from the CLAMP, cTAKES, and MetaMap experiments represent entities and concepts extracted by the tools that are not in the BM set. It is likely that some of the most frequent FP terms represent ASD-specific vocabulary due to their high frequency in the ASD texts. However, without thorough manual inspection of the context of these terms and comparison with a control (i.e. term frequencies in papers about ASD comorbidities such as ADHD and anxiety), it is unclear whether certain terms are specific to ASD or if they are associated with ASD comorbidities; some terms are closer to the DSM-5 characterization of ASD than others. Additionally, because of the heterogeneity of ASD, some predicted phenotypic traits can be more relevant to specific subgroups of ASD individuals than others. For example, language delay is a feature less associated with Asperger’s syndrome than perhaps other forms of ASD [16, 17]. Therefore, it would be helpful in the future to contextualize the terminology based on the sentences they appear in and cluster them around subgroups of ASD. Nonetheless, the predicted entities from this study, including the prioritized FP terms, could serve as a useful starting point for future studies seeking to develop and contextualize ASD terminology.

However, this study is not without limitations. One limitation of using the automatic rule-based labelling approach, even with a comprehensive list of ASD vocabulary, is the inability to perform word sense disambiguation (WSD). WSD is needed to differentiate ASD, as in autism spectrum disorder, from ASD, as in atrial septic defect, or toy *train* from spike *train*. WSD also presents a challenge to CLAMP, cTAKES, and MetaMap. CLAMP partially implements the clinical abbreviation recognition and disambiguation (CARD) framework [18] and also allows for a custom abbreviation list (which was not used in this study) [3]. However, all CLAMP predictions of “ASD” were mapped to the CUI for atrial septal defect. MetaMap implements WSD by favoring mappings that are semantically consistent with surrounding text [1]. It mapped “ASD” to the CUI for autism spectrum disorders (C1510586) 91.2% of the time in full-texts and 36.7% of the time in abstracts, where it is mapped to the CUI for pervasive developmental disorder (C0524528) 55.7% of the time. We were unable to use the word sense disambiguation module for cTAKES because it requires an additional database setup, however, it implements a similar approach as MetaMap. More research on WSD within the context of terminology for ASD is warranted.

Finally, the BM ASD terms we used do not represent a true gold standard, which caused the F1 scores to be relatively low for all three tools. Ideally, the gold standard ASD entities, which would include disease names, symptoms, behaviors, traits, etc., for the NER task should be comprehensive and labelled by human experts in the full-texts and abstracts. However, due to the difficulty, expensiveness, and time-consuming nature of this manual process, we used existing published ASD terms. We reasoned that as long as we compared CLAMP, cTAKES, and MetaMap to the same list of BM terms, the F1 score differences should reflect the tools’ relative performance difference in the NER task. While the BM terms are not comprehensive of ASD terminology, they represent a good starting point for automatically labelling a high volume of full-text articles and abstracts in a short period of time. However, we propose ways to expand the BM vocabulary by analyzing predictions from CLAMP, cTAKES, and MetaMap. Future studies can be done to consolidate the ASD terminology and their relationships, as well as relationships to different subgroups of ASD. A future direction that we are actively pursuing is to create an ASD ontology that can be used by clinicians to characterize patients with ASD, and can be used in predictive models to analyze free texts to aid ASD diagnosis.

## Conclusion

CLAMP has the best performance in terms of F1 score, and higher precision and slightly lower recall, compared to cTAKES and MetaMap. Also, CLAMP can predict longer chunks of text, which can be more descriptive of ASD. The preliminary ASD terms extracted from the PubMed literature in this study can be used to facilitate the precise diagnosis of ASD and improve our understanding of the phenotypic manifestations of the disorder. Future studies can be done to consolidate the ASD terminology by analyzing patients’ data, using methodology established by the current study, through collaborations with clinicians.

## Supplementary information

**Additional file 1**: Fig. 1. Frequencies of benchmark ASD terms. The frequencies of the benchmark ASD terms in A) 544 PubMed full-text articles and B) 20,408 PubMed abstracts are shown in three pie graphs. The top subgraph represents UMLS semantic type frequencies (T048 = Mental of Behavioral Dysfunction, T033 = Finding, T101 = Patient or Disabled Group, T041 = Mental Process, T054 = Social Behavior). The middle subgraph represents term frequencies for all BM terms. The term “general” represents generalized ASD characteristics and is expanded out in the bottom subgraph. The number in each section of the pie graph, which the size of the section is scaled to, represents the frequency as a percentage of all BM terms, and only percentages greater than 2 are labelled.

** Additional file 2**: Fig. 2. Frequency of FP terms predicted by CLAMP, cTAKES, and MetaMap. The frequencies of FP terms predicted by the three tools were combined and are shown as a pie graph. Prioritized CUIs represent the set of CUIs from CUI predictions shared by the three tools in the same sentence and co-occurring with a general BM term; non-prioritized CUIs represent all other CUIs. The normalized entity name for the CUI is displayed in square brackets beside the CUI. The number in each section of the pie graph, which the size of the section is scaled to, represents the frequency as a percentage of all FP predictions from CLAMP, cTAKES, and MetaMap combined, and only percentages greater than 2 are labelled.

**Additional file 3**: Please refer to the Excel file Supplemental_tables.xlsx.

## Data Availability

The code generated and datasets analyzed during the current study are available in the GitHub repository: https://github.com/WGLab/ASD_terminology.

## Abbreviations

ASD: Autism spectrum disorder
NLP: Natural language processing
UMLS: Unified medical language system
CUIs: Concept unique identifiers
BM: Benchmark
FP: False positive
NER: Named entity recognition
