# Supplementary figures and images for: Natural language processing (NLP) tools in extracting biomedical concepts from research articles: a case study on autism spectrum disorder
Source: BMC Med Inform Decis Mak. 2020 Dec 30;20(Suppl 11):322. doi: 10.1186/s12911-020-01352-2 (PMC7772897; doi:10.1186/s12911-020-01352-2)

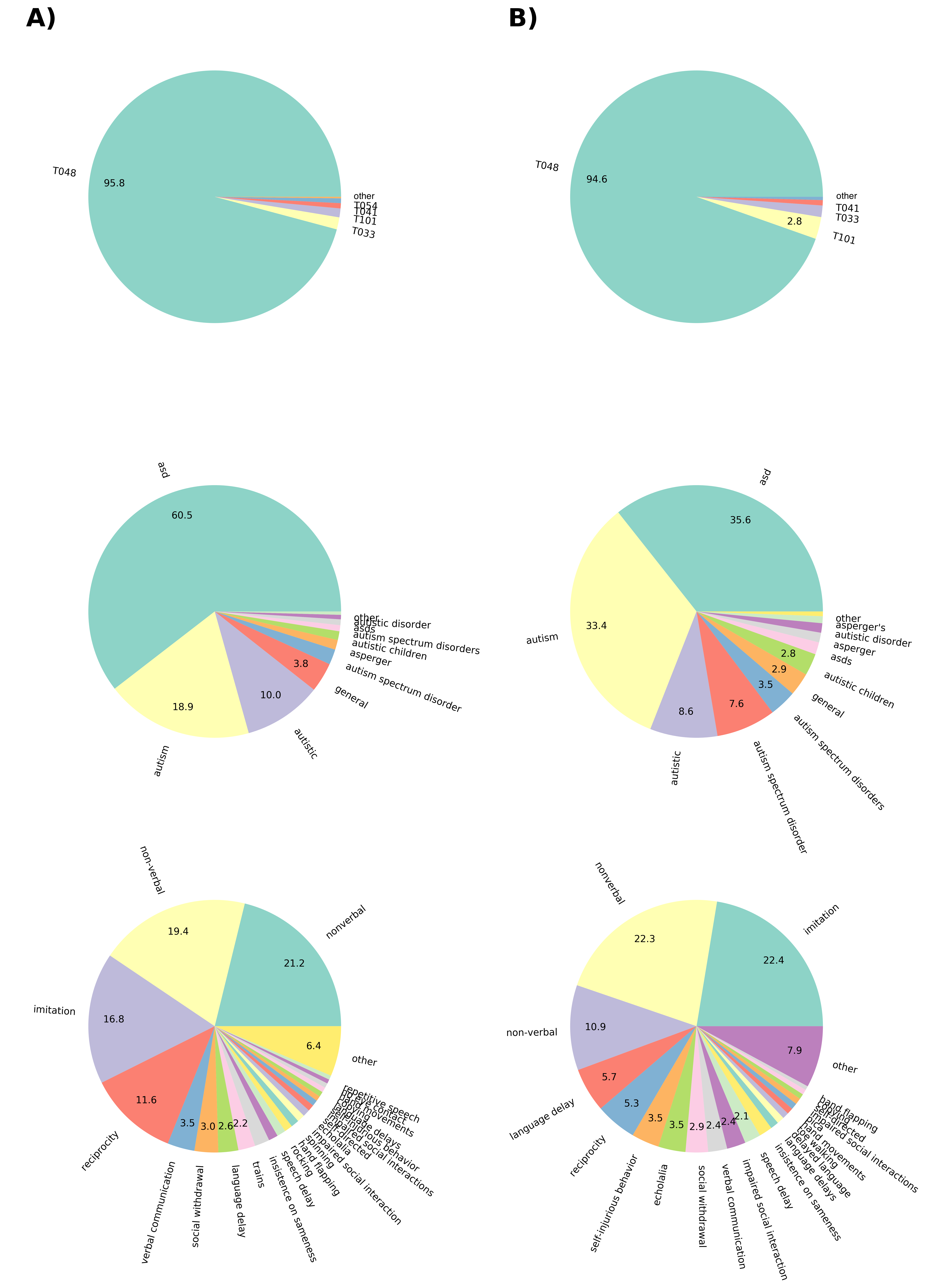

Supplement: Supplementary file 1 — Additional file 1: Fig. 1. Frequencies of benchmark ASD terms. The frequencies of the benchmark ASD terms in A) 544 PubMed full-text articles and B) 20,408 PubMed abstracts are shown in three pie graphs. The top subgraph represents UMLS semantic type frequencies (T048 = Mental of Behavioral Dysfunction, T033 = Finding, T101 = Patient or Disabled Group, T041 = Mental Process, T054 = Social Behavior). The middle subgraph represents term frequencies for all BM terms. The term “general” represents generalized ASD characteristics and is expanded out in the bottom subgraph. The number in each section of the pie graph, which the size of the section is scaled to, represents the frequency as a percentage of all BM terms, and only percentages greater than 2 are labelled. [file 12911_2020_1352_MOESM1_ESM.tiff]

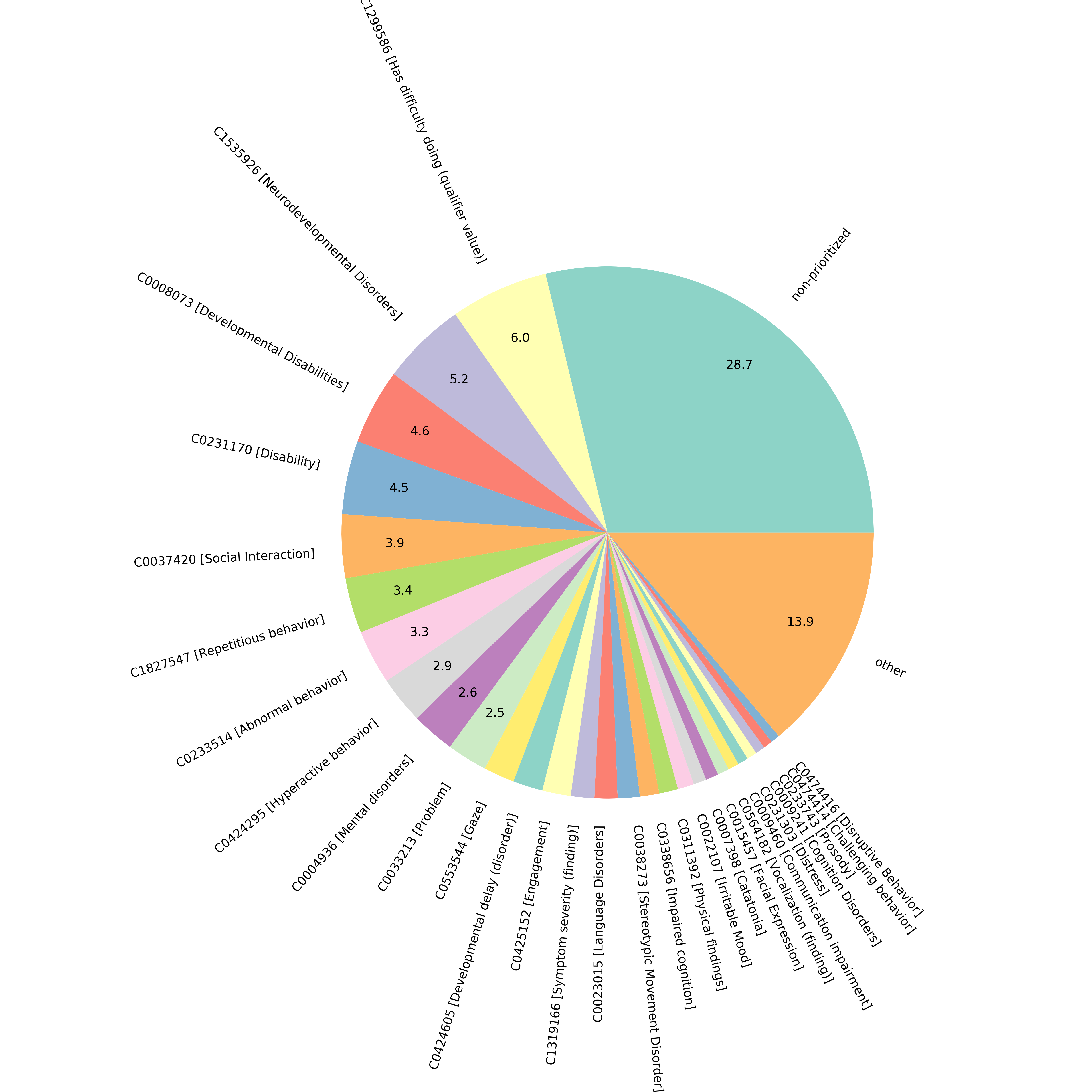

Supplement: Supplementary file 2 — Additional file 2: Fig. 2. Frequency of FP terms predicted by CLAMP, cTAKES, and MetaMap. The frequencies of FP terms predicted by the three tools were combined and are shown as a pie graph. Prioritized CUIs represent the set of CUIs from CUI predictions shared by the three tools in the same sentence and co-occurring with a general BM term; non-prioritized CUIs represent all other CUIs. The normalized entity name for the CUI is displayed in square brackets beside the CUI. The number in each section of the pie graph, which the size of the section is scaled to, represents the frequency as a percentage of all FP predictions from CLAMP, cTAKES, and MetaMap combined, and only percentages greater than 2 are labelled. [file 12911_2020_1352_MOESM2_ESM.png]
